# H_2_ Production from Formic Acid Using Highly Stable Carbon-Supported Pd-Based Catalysts Derived from Soft-Biomass Residues: Effect of Heat Treatment and Functionalization of the Carbon Support

**DOI:** 10.3390/ma14216506

**Published:** 2021-10-29

**Authors:** Jessica Alejandra Chaparro-Garnica, Miriam Navlani-García, David Salinas-Torres, Emilia Morallón, Diego Cazorla-Amorós

**Affiliations:** 1 Department of Inorganic Chemistry and Materials Institute, University of Alicante, 03080 Alicante, Spain; jessica.chaparro@ua.es (J.A.C.-G.); miriam.navlani@ua.es (M.N.-G.); 2 Department of Physical Chemistry and Materials Institute, University of Alicante, 03080 Alicante, Spain; david.salinas@ua.es

**Keywords:** soft-biomass, hydrothermal carbonization, N-doped porous carbon, Pd-based catalyst, formic acid dehydrogenation

## Abstract

The production of hydrogen from liquid organic hydrogen carrier molecules stands up as a promising option over the conventional hydrogen storage methods. In this study, we explore the potential of formic acid as a convenient hydrogen carrier. For that, soft-biomass-derived carbon-supported Pd catalysts were synthesized by a H_3_PO_4_-assisted hydrothermal carbonization method. To assess the impact of the properties of the support in the catalytic performance towards the dehydrogenation of formic acid, three different strategies were employed: (i) incorporation of nitrogen functional groups; (ii) modification of the surface chemistry by performing a thermal treatment at high temperatures (i.e., 900 °C); and (iii) combination on both thermal treatment and nitrogen functionalization. It was observed that the modification of the carbon support with these strategies resulted in catalysts with enhanced performance and outstanding stability even after six consecutive reaction cycles, thus highlighting the important effect of tailoring the properties of the support.

## 1. Introduction

Worldwide energy demand is continuously increasing due to the growth in world population, the economic and industrial growth of developing countries, as well as the living standards adopted by the developed countries. All this is negatively affecting the environmental problems that jeopardize the health of our planet and the life of humankind as we know it now. Concern about these issues has brought the search for alternative energy sources to the forefront of research. Among the explored alternatives, the use of hydrogen is very promising yet challenging. The main drawbacks that might overshadow the potential of hydrogen in the energy supply are mainly related to its difficult storage, the purity required for fuel cells, and safety concerns [1]. The deployment of hydrogen carrier molecules stands up as an auspicious option over the traditional storage systems [2,3,4]. Formic acid (FA, HCOOH), classified as a liquid organic hydrogen carrier (LOHC), has received great attention during the last decade [5,6,7,8,9,10,11].

The production of hydrogen from FA proceeds via a dehydrogenation reaction (HCOOH ↔ H_2_ + CO_2_) that can be promoted by both homogeneous [12,13] and heterogeneous [14,15,16] catalysts, the latter being the preferred choice because of advantages associated with their use. The heterogeneous catalysts used in this reaction are frequently based on Pd nanoparticles supported on materials of diverse composition [6,17,18,19,20,21,22], with carbon materials being some of the most common supports [7,23,24]. Despite the vast literature reporting on Pd-carbon-based catalysts for the dehydrogenation of FA, most of the studies are aimed at optimizing the properties of the catalytic active phase (i.e., particle size [23,25,26], composition [17,27,28,29,30], etc.), while the potential of tailoring the properties of the carbon support is frequently disregarded. The modification of the carbon materials that have been considered lies in the incorporation of nitrogen functional groups, which have been proven to both modify the properties of the metal active phases and participate actively in the dehydrogenation reaction [31,32,33,34].

We have previously observed that such a modification of the carbon support by introducing nitrogen atoms serves as a good strategy to obtain highly stable Pd-based catalysts for the production of hydrogen from FA [32]. However, aside from the incorporation of nitrogen species, very little has been discussed about other characteristics of the carbon supports that may also contribute to achieving enhanced catalytic performances. Beyond that observation, this study is addressed to further explore the potential of carbon materials-based catalysts in serving as catalytic support for the present application. With that in mind, a soft biomass residue was used to develop Pd-based catalysts by modulating the properties of the carbon support through three different strategies: (i) incorporation of nitrogen functional groups; (ii) modification of the surface chemistry by performing a thermal treatment at high temperatures (i.e., 900 °C); and (iii) combination of both heat treatment and nitrogen functionalization. The resulting carbon supports were loaded with Pd nanoparticles by following a simple protocol and the as-synthesized catalysts were assessed in the FA dehydrogenation reaction in the liquid phase. The developed materials showed excellent stability even after six consecutive cycles. The results indicated that not only the properties of the metal active phase but also the characteristics of the carbon supports are crucial in designing highly efficient catalysts for this reaction.

## 2. Materials and Methods

### 2.1. Activated Carbon-Based Supports Derived from Soft Biomass

The starting carbon support was synthesized by H_3_PO_4_-assisted hydrothermal carbonization using a soft-biomass waste following the procedure described elsewhere [35]. The as-prepared carbon support was named BC. To modify the surface chemistry of the carbon support, three strategies were followed: (i) Heat treatment was performed under a N_2_ atmosphere (80 mL min^−1^) using a heating rate of 5 °C min^−1^ up to 900 °C, maintaining this temperature for 15 min. The resulting heat-treated carbon was denoted as BC_TT. (ii) Modification of the surface chemistry by incorporating nitrogen functional groups using a protocol described elsewhere [35]. The N-containing support was denoted as N-BC. (iii) Combination of the above-mentioned heat treatment with nitrogen modification. The resulting carbon support was named as N-BC_TT.

### 2.2. Preparation of the Pd-Based Catalyst

Pd-based catalysts were synthesized by a wet impregnation method with Pd(OAc)_2_ as the metal precursor and suppressing the reduction step [32]. Briefly, the carbon support was dispersed in a specific volume of acetone, and an aqueous solution of 0.01 M Pd(OAc)_2_ was added to obtain Pd-based catalysts with a Pd content of 1 wt.%. Then, the mixture was stirred at room temperature. Finally, the as-prepared catalysts were washed with distilled water and dried at 60 °C overnight. The resulting catalysts were denoted as Pd/BC, Pd/BC_TT, Pd/N-BC, and Pd/N-BC_TT, for the N-free and N-containing catalysts, respectively. All the samples had a nominal metal content of 1 wt.%.

### 2.3. Characterization

The porous texture of all the carbon supports and catalysts was analyzed by physical N_2_ adsorption-desorption experiments at −196 °C in an automatic adsorption system (Micromeritics ASAP 2020 analyzer, Norcross GA, USA). Before the analysis, the samples were degassed at 200 °C for 6 h. The apparent surface area (S_BET_) and total micropore volume (V_DR_ N_2_) were calculated by applying the Brunauer–Emmett–Teller (BET) method and the Dubinin–Radushkevich (DR) equation, respectively, while the volume of mesopores was calculated from the difference between the volume of gas adsorbed at a relative pressure of 0.95 and the volume of micropores [36]. Pore size distributions were calculated from the 2D-NLDFT heterogeneous surface model using the SAIEUS software (Micromeritics, Norcross GA, USA) (available online at http://www.nldft.com/; accessed on 19 September 2021) [37].

X-ray photoelectron spectroscopy (XPS) was used to characterize the materials. N1s and Pd 3d (Pd 3d_5/2_ and Pd 3d_3/2_) spectra were analyzed. The equipment used to perform these measurements was a VG-Microtech Multilab 3000 spectrometer (ThermoFischer Scientific, Sussex, UK), and the deconvolution of the spectra was carried out by fitting the experimental data with a combination of Gaussian functions with a Lorentzian component and using a Shirley line to estimate the background signal. In addition, for the characterization of the surface chemistry of the activated carbon supports, temperature programmed desorption (TPD) measurements were carried in a DSC-TGA equipment (TA instruments, SDT Q600, New Castle, UK) coupled to a mass spectrometer (HiCube 80 Eco, Pfeiffer Vacuum, Asslar, Alemania). The activated carbons were heated at 950 °C under a helium flow of 100 mL min^−1^, using a heating rate of 20 °C min^−1^.

The pH at the point of zero charge, pH_PZC_, was also determined. The measurements were carried out by mixing a fixed amount of each activated carbon support (previously dried in an oven at 110 °C) with a fixed volume of ultrapure water and keeping the mixture under constant stirring for 24 h at 25 °C [38]. Afterward, the activated carbon was filtered, and the pH was measured. The equipment used for pH measurements was a pH meter, MM 374 Hach sensIONTM + multimeter.

The determination of the Pd average nanoparticle size was carried out by transmission electron microscopy (TEM) analysis, using a JEOL transmission electron microscope (JEM-2010, JEOL, Akishima, Japan) with a GATAN model ORIUS SC600 imaging camera assembled on the axis with the microscope on the bottom, integrated into the GATAN Digital Micrograph 1.80.70 imaging and acquisition program for GMS 1.8.0. and ImageJ software. The samples were prepared by sonicating a little amount of catalyst in ethanol for a few minutes. Subsequently, a drop of the suspension was deposited on a copper TEM grid with Lacey carbon film. To determine the dispersion (D (%)) of the Pd nanoparticles, a spherical geometry was assumed [39]. Inductively coupled plasma-optical emission spectroscopy (ICP-OES) was used to determine the Pd content in each catalyst. This measurement was performed with a Perkin-Elmer Optima 4300 system (Perkin Elmer, Waltham, MA, USA.

### 2.4. Catalytic Tests

The developed catalysts were assessed in the dehydrogenation of FA in the liquid phase while keeping the reaction temperature at 75 °C. The tests were performed with 0.15 g of catalyst and an aqueous solution of FA and sodium formate (SF) with a molar ratio of 9 to 1 and a final concentration of 1 M. As-prepared Pd-based catalyst was dispersed in 5 mL of distilled water in the reactor, which was connected to a burette system with a graduated glass tube. After purging the system, 5 mL of an aqueous solution of FA and sodium SF was incorporated into the reactor to achieve the above-mentioned concentration. The gas generated was monitored by registering the water displacement. In order to evaluate the stability of the catalysts, six consecutive reaction cycles were carried out under identical experimental conditions. The spent catalyst was recovered from the reactor by filtration after each cycle. It is important to mention that a loss of catalyst mass might take place after each reaction cycle during the filtration step. In this study, the estimated loss of catalyst mass was between 6 and 10% in each reaction cycle. The recovered catalyst was used in the next reaction cycle without drying.

The following equation was used to calculate TOF values (h^−1^):

$$\mathrm{TOF}\;\left(h^{-1}\right)=\frac{{\mathrm{produced}\;H}_{2}\;\left(\mathrm{mole}\right)}{\mathrm{Pd}\;\mathrm{atoms}\;\left(\mathrm{mole}\right)\times \mathrm{time}\;\left(h\right)}$$

where the produced H_2_ (mole) is the mole of H_2_ obtained from the third reaction run (after 4 min of reaction), and Pd atoms corresponds to the actual Pd content obtained by ICP-OES analysis. Concerning TOF values based on surface Pd atoms, these were normalized by surface Pd atoms instead of the total Pd. The surface Pd atoms were calculated from Pd nanoparticle dispersion (D (%)) by using the following equation:

$$\mathrm{Surface}\;\mathrm{Pd}\;\mathrm{atoms}=\frac{{\mathrm{mole}}_{\mathrm{Pd}}\cdot D\left(\%\right)}{100}$$

The nanoparticle dispersion was estimated by assuming spherical nanoparticle geometry and using the following equation:

$$D\left(\%\right)=10^{21}\frac{6{M\rho }_{\mathrm{site}}}{\rho_{\mathrm{Pd}}{\mathrm{Nd}}_{\mathrm{TEM}}}$$

where M is the atomic weight, N is the Avogadro’s number and d_TEM_ is the average diameter of the nanoparticles, while ρ_site_ and ρ_Pd_ are the Pd surface site density and the metal density, respectively.

## 3. Results

**Figure 1:**
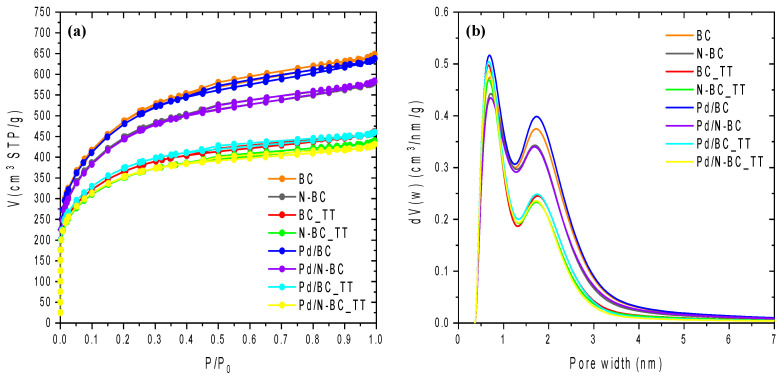
N_2_ adsorption–desorption isotherms at −196 °C of carbon supports and Pd-based catalysts (**a**) and their pore size distribution (**b**).

Figure 1 shows the adsorption–desorption isotherms of N_2_ at −196 °C (Figure 1a) and the pore size distribution profiles calculated by NLDFT (Figure 1b) of the carbon supports derived from soft biomass and the counterpart Pd-based catalysts studied. Table 1 shows the textural properties determined from the N_2_ adsorption–desorption isotherms. As can be seen, all isotherms have large N_2_ uptakes at low relative pressures, which are characteristic of microporous solid (type I isotherm). Moreover, a hysteresis loop can be seen, which is related to the presence of mesopores, indicating that these isotherms correspond to a combination of type I and IV isotherms, according to the IUPAC classification [36]. Regarding the pore size distributions (PSDs), the profiles display a bimodal distribution with a first peak at around 0.8 nm and a region of pore sizes higher than 2 nm, arising from the presence of mesopores. The heat treatment performed on the carbon supports (BC_TT and N-BC_TT samples) leads to a decrease in the adsorption capacity compared to BC sample as a consequence of the shrinkage of the porosity (Figure 1 and Table 1). However, it was also observed in all cases that neither the introduction of nitrogen functional groups nor the incorporation of Pd nanoparticles modified considerably the porous texture of the corresponding starting activated carbon (Figure 1 and Table 1).

**Figure 2:**
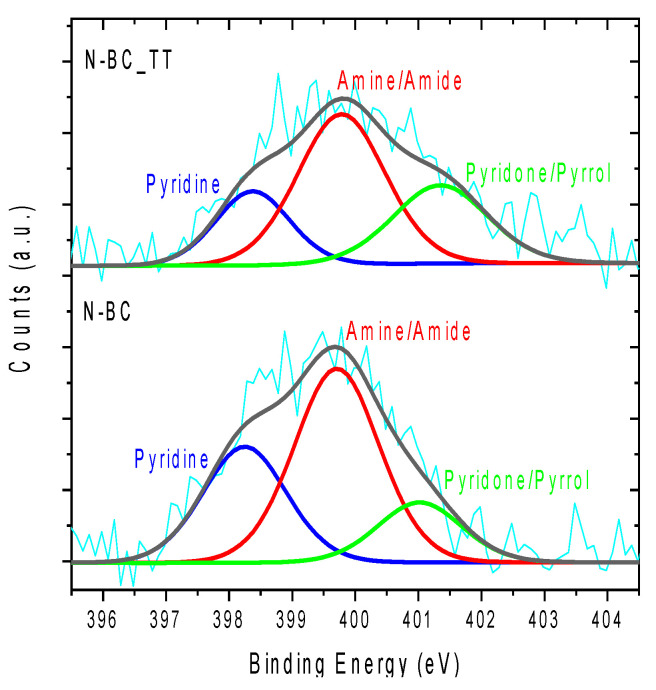
XPS N1s spectra of the N-BC and N-BC_TT.

**Figure 3:**
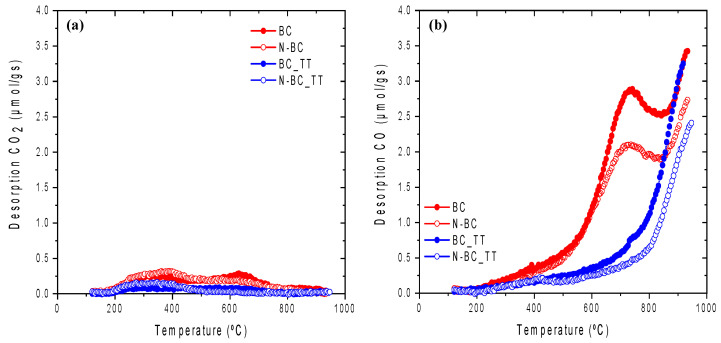
TPD profiles for all carbon supports (**a**) CO_2_-evolving groups and (**b**) CO-evolving groups.

Concerning the surface chemistry of the activated carbons supports, Figure 2 shows the XPS spectra of the N1s of the functionalized activated carbons supports, which are deconvoluted into several peaks related to the different nitrogen groups. From the XPS data (see Table 2), it was observed that a very similar surface nitrogen content was incorporated in both the raw support and the heat-treated counterpart (i.e., 1.7 and 1.3 at. % for N-BC and N-BC_TT, respectively). Table 2 also includes the quantification of oxygen groups from TPD measurements of all activated carbons supports (See Figure 3). According to these results, a decrease in the amount of oxygen is observed after nitrogen functionalization, which is in agreement with previous studies [40,41]. It is important to highlight that the decrease in oxygen functional groups is mainly related to CO-evolving groups (phenol and carbonyl groups) (see Figure 3) through which N-functional groups incorporation occurs.

The assignment of the deconvoluted peaks of the different nitrogen functional groups present in N-BC and N-BC_TT supports was carried out according to the literature [42,43,44]. As can be seen in Figure 2, the spectra were deconvoluted into three peaks at 398.3, 399.7, and 401.2 eV, assigned to imines/pyridines, amines/amides, and pyridone/pyrrole, respectively [40,45,46]. The heat treatment of the carbon material slightly changed the relative proportion of N-species incorporated in the support. However, the chemical nature of the nitrogen functional groups incorporated in the post-treatment were not altered with respect to the original BC support. Figure S6 (Supplementary materials) shows the XPS spectra of the N 1s of the BC and BC_TT supports in which the absence of nitrogen in these materials is confirmed.

Figure 3 shows the CO_2_ and CO TPD profiles for all activated carbons supports. TPD data in Table 2 indicates that N functionalization occurs through reaction with oxygen functional groups, mainly with CO-type groups which are the most abundant. It can be seen that BC has CO-evolving groups at temperatures lower than 600 °C. Also, BC is rich in phenol groups, which evolve as CO between 600 °C and 700 °C, and in carbonyl groups that are desorbed at around 800 °C [47,48,49]. The CO-evolving groups at lower temperatures can be attributed to the presence of anhydride groups that decompose forming one CO and one CO_2_ molecule. Regarding the CO_2_ profile, there is small CO_2_ desorption between 200 °C and 450 °C that can be assigned to the presence of carboxylic groups and anhydrides, and the desorption at higher temperatures can be due to lactones decomposition [47,49].

After functionalization (N-BC), a significant decrease in the evolution of CO was observed at high temperatures, which indicates that during the functionalization reaction phenol and carbonyl groups are consumed, confirming that the generation of nitrogen groups occurs through substitution reactions with these oxygen groups [35,50].

The profiles corresponding to the heat-treated activated carbon support (BC_TT) show an important decrease in the amount of CO-type groups compared to the pristine activated carbon (BC). As in the case of the non-treated support, the nitrogen functionalization (N-BC_TT) produces a decrease in the CO-type groups, confirming that the substitution reactions occur through this type of group.

To check the acid–base character of the different activated carbons supports, the measurement of the pH at the point of zero charge (pH_PZC_) was carried out and the results are listed in Table 2. As expected, the pristine activated carbon support (BC) had an acid character with a pH_PZC_ value of 4.8, which is related to the small amount of CO_2_-type groups and to the possible presence of some residual phosphorus-containing groups. The increase in the pH_PZC_ after the thermal treatment (BC_TT) is a consequence of the removal of the most acidic CO_2_-type groups and weakly acidic CO-type groups. The functionalization with N groups produces a small increase in pH that can be explained by the basic character of the incorporated N groups.

**Figure 4:**
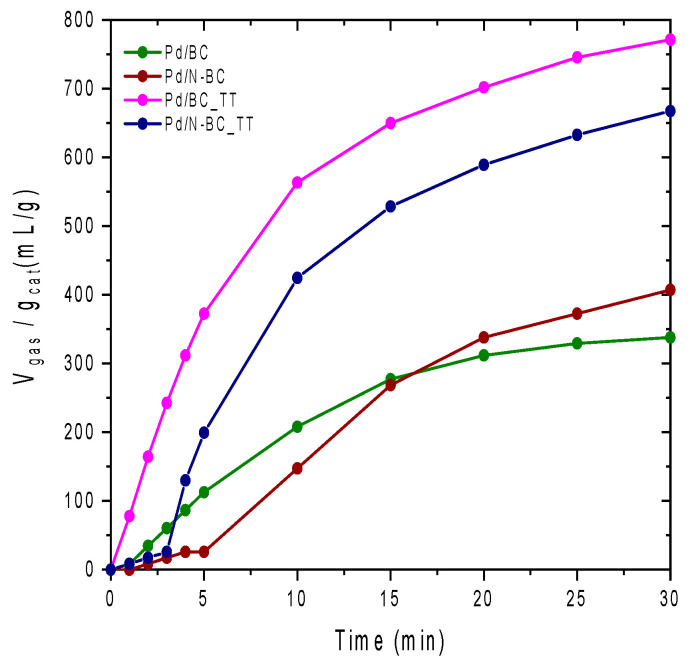
Gas evolution profiles (H_2_ + CO_2_) attained by Pd/BC, Pd/BC_TT, Pd/N-BC, and Pd/N-BC_TT in the first reaction cycle.

As for the results of the catalytic tests, Figure 4 depicts the total volume of gas (H_2_ + CO_2_) generated per gram of catalyst attained by Pd/BC, Pd/BC_TT, Pd/N-BC, and Pd/N-BC_TT catalysts in the first reaction cycle.

As can be seen in Figure 4, for those N-free samples the reaction proceeds smoothly just after the addition of the FA/SF mixture in the reaction medium, while an induction time is needed in the N-containing counterparts. Since the nanoparticles were not pre-reduced for any of the catalysts, the difference displayed in the first minutes of the reaction might be related to the different Pd reducibility under reaction conditions. The induction time required for Pd/N-BC and Pd/N-BC_TT suggests that the reduction of Pd species is somehow delayed in these samples compared to the N-free counterpart, and the hydrogen generated during the first minutes of the reaction is used in the in situ reduction of the Pd precursor to Pd nanoparticles. Such observation is in good agreement with previous studies that pointed out the role of N-atoms in stabilizing Pd^2+^ species [51,52]. In contrast, according to the observed profiles, the reduction of Pd species to form Pd nanoparticles was less impeded in N-free catalysts, which directly results in the sharp production of gas registered for these samples during the first reaction minutes. Especially, Pd/BC_TT showed an extraordinary initial reaction rate compared to the non-treated counterpart catalyst (20 and 81 mL_gas_.min^−1^.g_cat_^−1^ for Pd/BC and Pd/BC_TT, respectively), which suggests the role of the thermal treatment in modifying the catalytic activity of the assessed samples. In this line, it was seen that, even though Pd was loaded onto BC_TT and BC under identical experimental conditions, and both Pd/BC_TT and Pd/BC catalysts had nearly the same Pd content (0.73 and 0.80 wt.%, respectively, vide infra), these catalysts might have different properties related to both metal species and support. As for the metal species, TEM analysis (vide infra) indicated that small Pd nanoclusters (or well-dispersed Pd species) were formed in Pd/BC_TT catalyst just after the impregnation of the support with the Pd precursor, which suggests that there was a strong metal–support interaction existing in this case, while such nanoclusters were not detected for any of the other fresh catalysts. According to the activity shown by Pd/BC_TT, it seems that such small Pd clusters are highly active in the decomposition of FA.

Concerning the properties of the support, it was seen from the TPD analysis and pH_PZC_ that the surface chemistry of BC was modified by the thermal treatment performed (BC_TT). According to that analysis, the oxygen surface groups present in the pristine support were significantly reduced after the thermal treatment (3764 and 1597 µmol g^−1^ of O in BC and BC_TT, respectively), which is directly related to the decrease in the surface acidic groups. These results are in good agreement with the increase in pH_PZC_ from 4.8 for the pristine support (BC) to 6.6 for the heat-treated counterpart (BC_TT). Thus, the surface of the Pd/BC_TT catalyst has a less acidic character than that of Pd/BC counterpart, which is expected to favor the interaction with the FA molecules, thus having a positive effect on the catalytic performance.

As expected, the incorporation of N functional groups increased the pH at the point of zero charge compared to the N-free counterpart supports and, even though there is not a straightforward relationship between that value and the catalytic activity of the studied samples, those catalysts with a less acidic surface showed better activity (See Table 2 and Figure 4). In this line, the total volume of gas generated after 30 min of reaction per gram of catalysts followed the order Pd/BC < Pd/N-BC < Pd/N-BC_TT < Pd/BC_TT, with a total volume of gas per gram of catalyst of 338, 407, 667, and 771 mL_gas_.g_cat_^−1^, respectively. The comparison of the catalysts based on non-heat-treated supports (Pd/BC and Pd/N-BC) is in good agreement with previous studies, in which the beneficial effect of N-containing carbon-based catalysts in enhancing the catalytic performance towards the dehydrogenation of FA has been shown [31,32,33].

Aside from the effect of N-functional groups, the thermal treatment of the carbon supports also had a significant effect in enhancing the ability of the catalysts to boost the dehydrogenation of FA. In this regard, the N-free catalysts showed an enhancement of ~128%, in terms of the total volume of gas generated after 30 min of reaction per gram of catalyst (338 and 771 mL_gas_.g_cat_^−1^, for Pd/BC and Pd/BC_TT, respectively), while N-containing catalysts displayed an enhancement of ~64% (407 and 667 mL_gas_.g_cat_^−1^, for Pd/N-BC and Pd/N-BC_TT, respectively), thus suggesting the great potential of the strategy tackled in achieving catalysts with tailored performance towards the dehydrogenation of FA.

**Figure 5:**
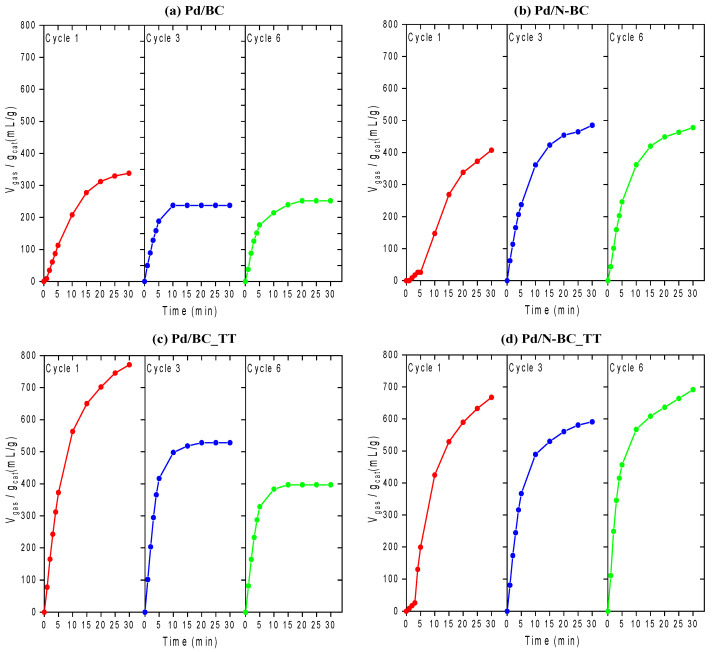
Gas evolution profiles (H_2_ + CO_2_) achieved for the 1st, 3rd, and 6th cycle for: (**a**) Pd/BC, (**b**) Pd/N-BC, (**c**) Pd/BC_TT, and (**d**) Pd/N-BC_TT.

The catalytic performance of the developed materials was further assessed by performing six consecutive reaction runs. The results of such stability tests are plotted in Figure 5. Figure S1 shows the gas evolution profiles (H_2_ + CO_2_) achieved for all cycles of each catalyst.

The gas evolution profiles obtained demonstrate that all the catalysts assessed displayed great stability even after six consecutive reaction runs at 75 °C, which is a significant breakthrough achieved by the materials developed in this study over some other Pd-based catalysts reported elsewhere which showed significant catalytic activity decay even after few catalytic cycles [28,53,54,55,56].

As indicated by these profiles, most of the samples preserved a good activity even after six consecutive runs. In particular, Pd/N-BC and Pd/N-BC_TT displayed good activity and outstanding stability during the six cycles of reaction, which points out the suitability of the developed materials and highlights the key role of nitrogen functionalization in attaining promising catalysts for the dehydrogenation of FA. Among those investigated, the Pd/N-BC_TT sample is the most promising catalyst, since, despite the better activity shown by the N-free counterpart catalysts in the first cycle, its activity partially decayed along with the cycles, while that of Pd/N-BC_TT was preserved. This indicated the potential of tuning the surface chemistry of the carbon support in attaining enhanced catalysts for the dehydrogenation of FA. Previous studies had already pointed out the role of nitrogen functionalization and/or nitrogen incorporation in the carbon supports [31,34,57,58], while the potential of further modifying the properties of the support and its chemical surface has not been explored. This study may pave the way for new works addressing the impact of tuning the surface chemistry of carbon-based catalysts.

**Figure 6:**
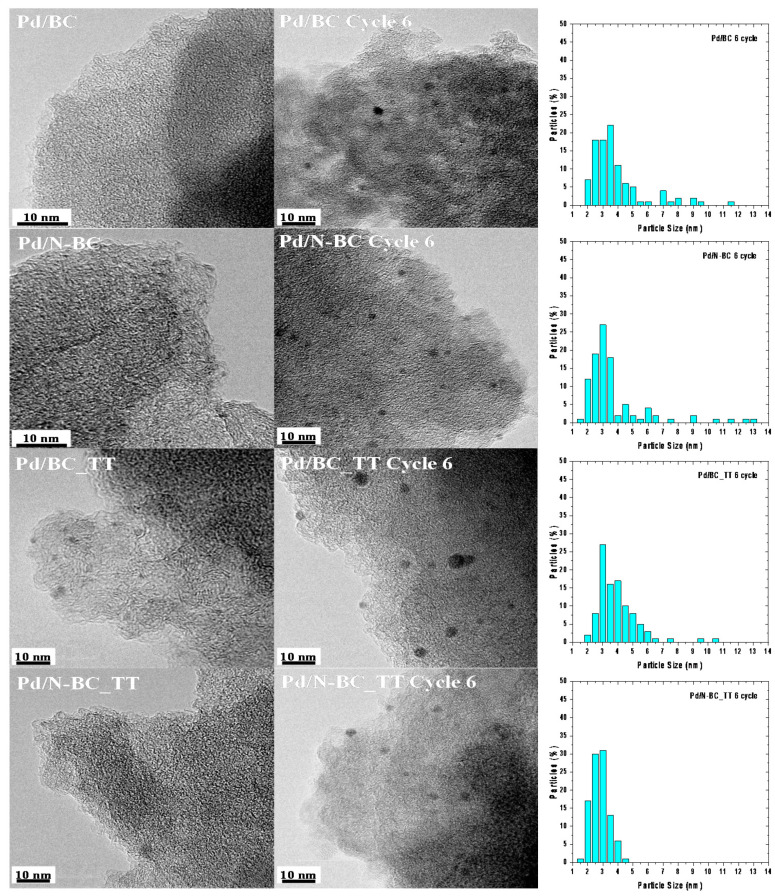
TEM micrographs for catalyst fresh and used after six cycles, and histograms with Pd nanoparticle size distributions for the used catalysts.

The great stability of the developed materials is backed up by the properties of the spent catalysts. Figure 6 shows the TEM micrographs registered for the fresh and spent samples, and Table 3 lists the average nanoparticle size (d_TEM_) determined by TEM, the dispersion of the nanoparticles (D (%)), and the Pd content for the fresh and spent catalysts. Additionally, Figures S2–S5 show more TEM micrographs of both fresh and used Pd/BC, Pd/N-BC, Pd/BC_TT, and Pd/N-BC_TT catalysts, respectively. As was previously mentioned, none of the fresh catalysts showed the presence of well-defined nanoparticles, but small clusters can be identified in Pd/BC_TT catalysts, which might suggest the better interaction between the metal precursor and BC_TT support and the higher reducibility character of the heat-treated support. Such clusters might be also responsible for the outstanding behavior of Pd/BC_TT in the first catalytic run. The micrographs of the spent catalysts reveal that, in all cases, well-defined and dispersed Pd nanoparticles were formed in situ under reaction conditions, which had an average nanoparticle size of 3.6, 3.5, 3.7, and 2.6 nm for the spent Pd/BC, Pd/N-BC, Pd/BC_TT, and Pd/N-BC_TT catalysts, respectively. It must be noted that the lowest average particle size and the narrowest particle size distribution was obtained for the sample Pd/N-BC_TT, which was prepared over the heat-treated support (with the lowest amount of surface oxygen groups) and subsequently modified with N-functional groups. Also, ICP confirmed that there was no significant leaching of Pd after six reaction runs. These results demonstrate the stability of the Pd active phase, which did not experience either sintering or leaching after performing six consecutive cycles at 75 °C.

**Figure 7:**
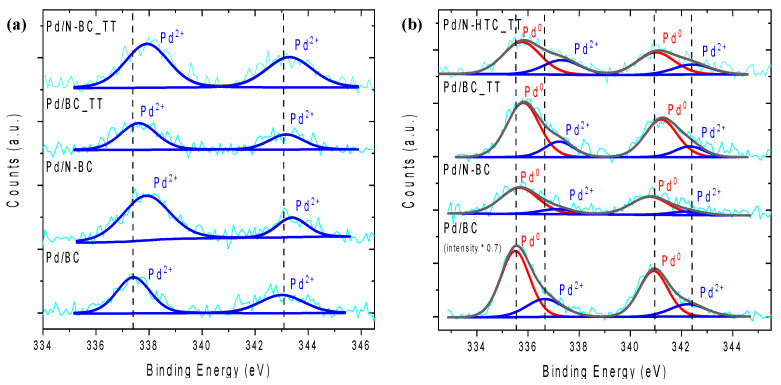
XPS Pd 3d spectra of (**a**) fresh catalysts and (**b**) after six cycles.

Concerning the electronic properties of the metal nanoparticles present in the catalysts, Figure 7 includes the XPS Pd 3d spectra for the four fresh and spent catalysts. It was observed that both Pd^0^ and Pd^2+^ species were present in all the spent samples, while only Pd^2+^ was detected in the fresh counterparts. As previously observed, both Pd^0^ and Pd^2+^ species are important in the reaction. The simplified mechanism involves the following three steps [17]: (i) adsorption of formate ions on the surface of Pd nanoparticles favored by the presence of electron-deficient Pd species; (ii) cleavage of the C−H bond of the adsorbed formate ion boosted by electron-rich Pd species (considered as the rate-limiting step in the literature); (iii) release of H_2_ and the regeneration of the catalyst.

It should be mentioned that Pd-based catalysts prepared from nitrogen-containing support show a shift towards higher binding energies in XPS Pd 3d spectra compared with catalysts based on nitrogen-free carbon supports, which was assigned to the Pd–N interaction. This is in good agreement with TEM micrographs and again demonstrates the successful reduction of the metal precursor in the reaction medium. It is important to notice that the methodology used suppresses the addition of reducing additives, such as NaBH_4_, which are frequently used in the preparation of metal nanoparticles-based catalysts, thus reducing the time, cost, and environmental impact of the process.

As for the initial TOF values achieved in the third reaction run, in which no induction time was needed for any of the samples, they were calculated to be 670, 892, 1654, and 1474 h^−1^, for Pd/BC, Pd/N-BC, Pd/BC_TT, and Pd/N-BC_TT, respectively, expressed based on the total Pd atoms. A more suitable comparison can be done considering the Pd surface atoms. The initial TOF values based on Pd surface atoms achieved after the 6^th^ reaction cycle were of 2705, 3433, 5435, and 5593 h^−1^ for Pd/BC, Pd/N-BC, Pd/BC_TT, and Pd/N-BC_TT, respectively, again highlighting the great potential of the approach reported herein for the preparation of carbon-based catalysts with tailored surface chemistry properties. Those TOF values are higher than those obtained in the first reaction cycle by other carbon-supported catalysts reported elsewhere (i.e., Pd@CN catalysts with mesoporous carbon nitride, TOF of 49.8 h^−1^ [59]; Pd/C nanocatalyst, TOF of 228.3 h^−1^ [60]; AuPd–MnO_x_ nanocomposite immobilized on ZIF-8 reduced graphene oxide (ZIF-8–rGO), TOF of 382 h^−1^ [53]; Carbon-supported Pd nanoparticles, TOF of 835 h^−1^ [61]; Pd particles uniformly embedded in N-enriched mesoporous carbon, TOF of 913 h^−1^ [62]; Pd nanoparticles confined in carbon nanotubes, TOF of 1135 h^−1^ [63], etc.).

## 4. Conclusions

A series of soft-biomass-derived carbon-supported Pd catalysts were synthesized by a simple and straightforward methodology. Their performance in the dehydrogenation of FA in the liquid phase was evaluated by checking the effect of the properties of the supports. Three strategies were followed: (i) incorporation of nitrogen functional groups; (ii) modification of the surface chemistry by performing a thermal treatment at high temperatures; and (iii) combination of both thermal treatment and nitrogen functionalization. The modification of the surface chemistry of the support by removing oxygen functional groups and introducing nitrogen functional groups resulted in materials with less surface acidity and the presence of basic N functional groups, that favor their interaction with the FA molecules. The resulting Pd-based catalysts displayed good activity and excellent stability even after six reaction cycles. It was observed that the spent catalysts did not show either significant aggregation of the metal nanoparticles or metal leaching, which points out the stability of the active metal phases. The approach considered in this study may pave the way for the design of efficient catalysts for the dehydrogenation of FA by modulating the surface chemistry of the carbon-based supports.

## Figures and Tables

**Table 1:** Porous texture characterization.

Sample	S_BET_ (m^2^ g^−1^)	V_DR_ N_2_ (cm^3^ g^−1^)	V_meso_ N_2_ (cm^3^ g^−1^)
BC	1780	0.71	0.27
N-BC	1600	0.68	0.20
BC_TT	1330	0.54	0.16
N-BC_TT	1240	0.52	0.14
Pd/BC	1730	0.70	0.27
Pd/N-BC	1590	0.63	0.26
Pd/BC_TT	1325	0.53	0.17
Pd/N-BC_TT	1245	0.53	0.13

**Table 2:** Surface chemistry of the soft-biomass derived carbon supports.

Sample	N _XPS_ (at. %)	CO_2 TPD_ (µmol g^−1^)	CO _TPD_ (µmol g^−1^)	O _TPD_ (µmol g^−1^)	pH_PZC_
BC	-	373	3018	3764	4.8
N-BC	1.7	373	2390	3136	5.6
BC_TT	-	122	1353	1597	6.6
N-BC_TT	1.3	132	1087	1351	7.1

**Table 3:** Results of TEM and ICP analysis.

Catalyst	d_TEM_ (nm)	D (%)	Pd Loading wt.% (ICP)
Pd/BC	-	-	0.80
Pd/BC Cycle 6	3.6 ± 1.8	25.0	0.73
Pd/N-BC	-	-	0.76
Pd/N-BC Cycle 6	3.5 ± 2.2	25.7	0.75
Pd/BC_TT	-	-	0.73
Pd/BC_TT Cycle 6	3.7 ± 1.3	24.3	0.71
Pd/N-BC_TT	-	-	0.70
Pd/N-BC_TT Cycle 6	2.6 ± 0.9	34.6	0.70

## Data Availability

Not applicable.

## Supplementary Materials

The following are available online at https://www.mdpi.com/article/10.3390/ma14216506/s1, Figure S1. Gas evolution profiles (H_2_+CO_2_) achieved for the all cycles for: (a) Pd/BC, (b) Pd/N-BC, (c) Pd/BC_TT, and (d) Pd/N-BC_TT, Figure S2. TEM micrographs for Pd/BC catalyst fresh and used after 6 cycles, Figure S3. TEM micrographs for Pd/N-BC catalyst fresh and used after 6 cycles, Figure S4. TEM micrographs for Pd/BC_TT catalyst fresh and used after 6 cycles, Figure S5. TEM micrographs for Pd/N-BC_TT catalyst fresh and used after 6 cycles, Figure S6. XPS N1s spectra of the BC and BC_TT.
