# Supplementary material for: H2 Production from Formic Acid Using Highly Stable Carbon-Supported Pd-Based Catalysts Derived from Soft-Biomass Residues: Effect of Heat Treatment and Functionalization of the Carbon Support
Source: Materials (Basel). 2021 Oct 29;14(21):6506. doi: 10.3390/ma14216506 (PMC8585402; doi:10.3390/ma14216506)
Supplement: Supplementary file 1 [file materials-14-06506-s001.zip › materials-1432242-supplementary.pdf]

materials

MDPI

Supplementary Materials

H_2 Production from Formic Acid Using Highly Stable Carbon-Supported Pd-Based Catalysts Derived from Soft-Biomass Residues: Effect of Heat Treatment and Functionalization of the Carbon Support

Jessica Alejandra Chaparro-Garnica [1], Miriam Navlani-García [1], David Salinas-Torres [2], Emilia Morallón [2] and Diego Cazorla-Amorós [1,*]

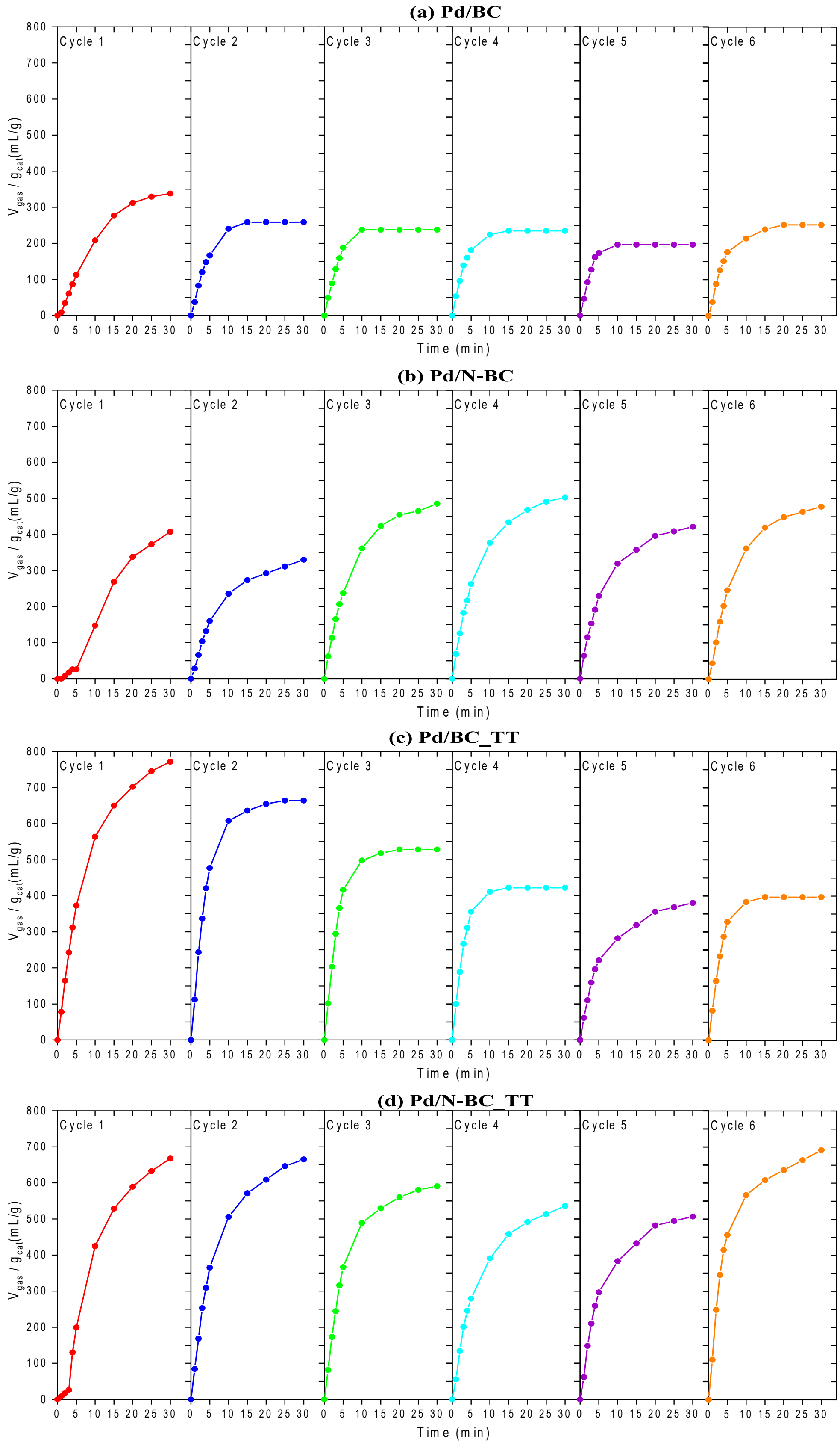

Figure S1. Gas evolution profiles (H_2+CO_2) achieved for the all cycles for: (**a**) Pd/BC, (**b**) Pd/N-BC, (**c**) Pd/BC_TT, and (**d**) Pd/N-BC_TT.

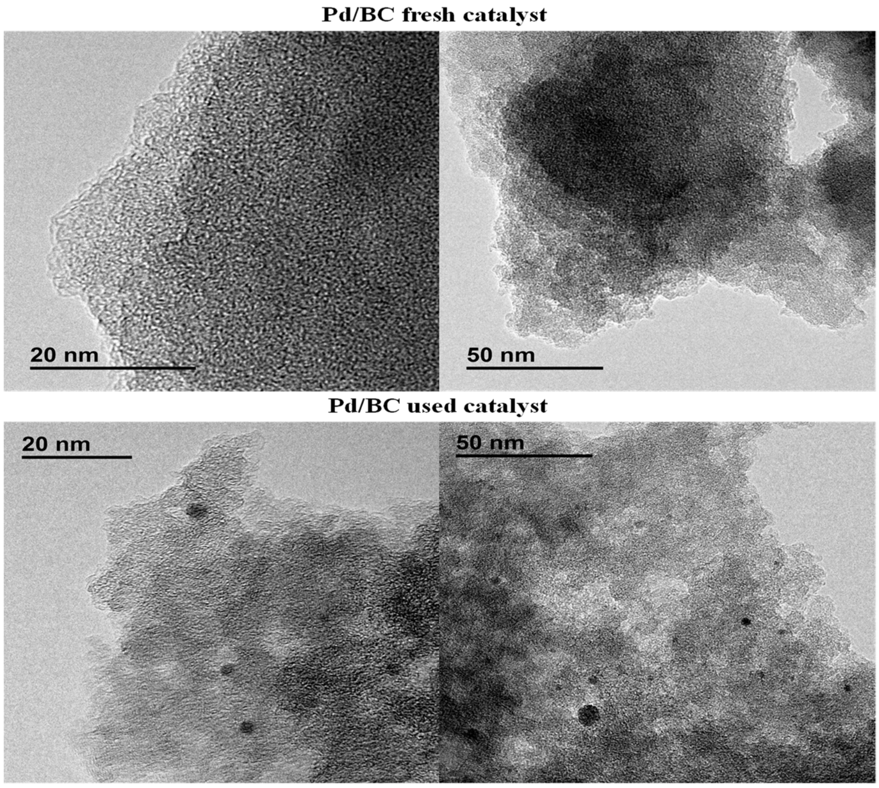

Figure S2. TEM micrographs for Pd/BC catalyst fresh and used after 6 cycles.

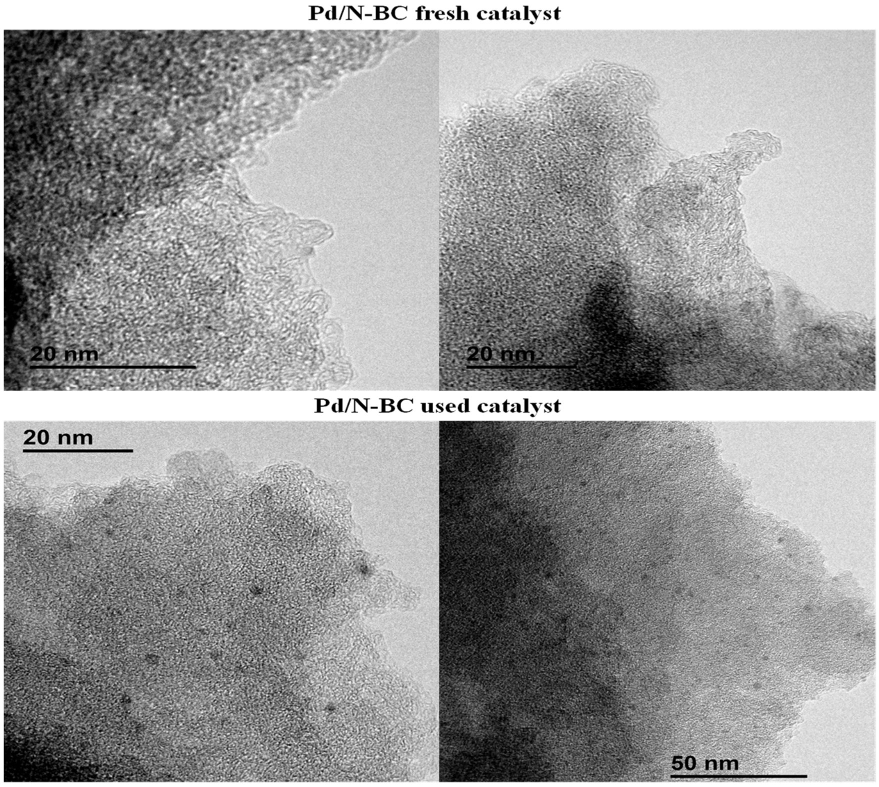

Figure S3. TEM micrographs for Pd/N-BC catalyst fresh and used after 6 cycles.

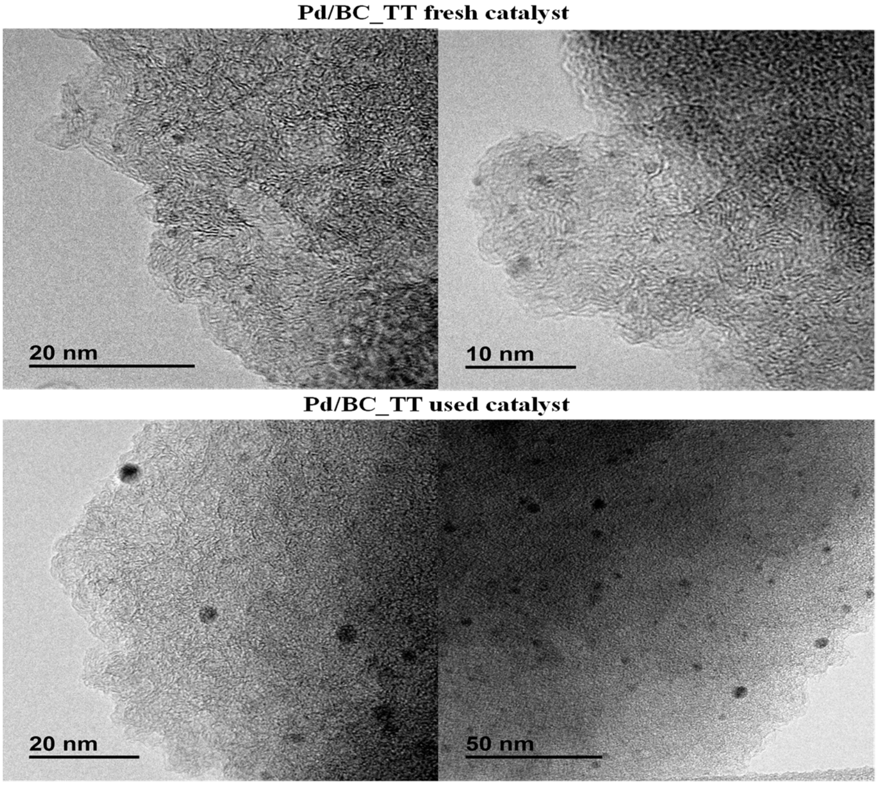

Figure S4. TEM micrographs for Pd/BC_TT catalyst fresh and used after 6 cycles.

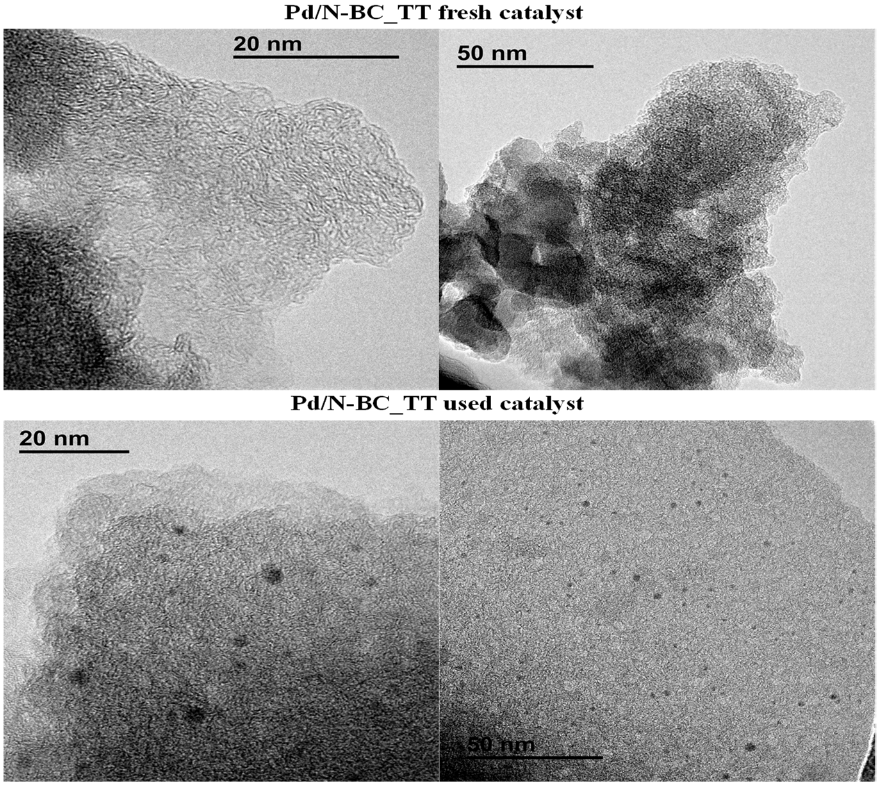

Figure S5. TEM micrographs for Pd/N-BC_TT catalyst fresh and used after 6 cycles.

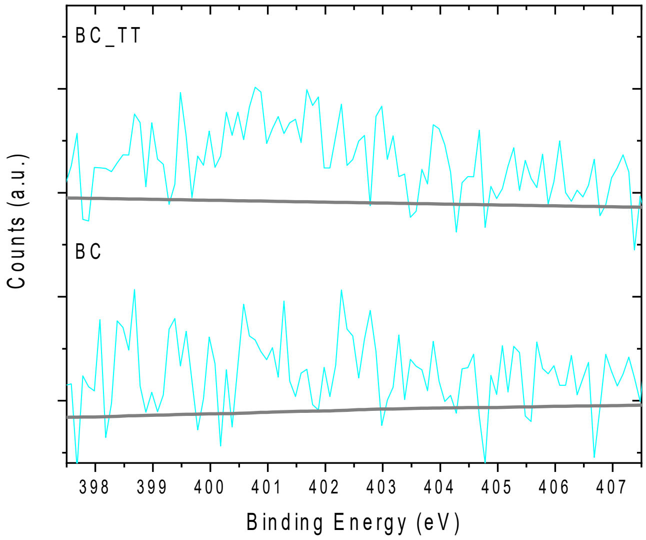

Figure S6. XPS N1s spectra of the BC and BC_TT.
